# Origin of the enhanced Nb_3_Sn performance by combined Hf and Ta doping

**DOI:** 10.1038/s41598-021-97353-w

**Published:** 2021-09-08

**Authors:** Chiara Tarantini, Fumitake Kametani, Shreyas Balachandran, Steve M. Heald, Laura Wheatley, Chris R. M. Grovenor, Michael P. Moody, Yi-Feng Su, Peter J. Lee, David C. Larbalestier

**Affiliations:** 1 National High Magnetic Field Laboratory, Florida State University, Tallahassee, FL 32310 USA; 2 Department of Mechanical Engineering, FAMU-FSU College of Engineering, Florida State University, Tallahassee, FL 32310 USA; 3 Advanced Photon Source, Argonne National Laboratory, Argonne, IL 60439 USA; 4 Department of Materials, Oxford University, Parks Road, Oxford, OX1 3PH UK; 5 Now at Materials Science and Technology Division, Oak Ridge National Laboratory, Oak Ridge, TN 37831 USA

**Keywords:** Superconducting properties and materials, Superconducting properties and materials

## Abstract

In recent years there has been an increasing effort in improving the performance of Nb_3_Sn for high-field applications, in particular for the fabrication of conductors suitable for the realization of the Future Circular Collider (FCC) at CERN. This challenging task has led to the investigation of new routes to advance the high-field pinning properties, the irreversibility and the upper critical fields (*H*_*Irr*_ and *H*_*c2*_, respectively). The effect of hafnium addition to the standard Nb-4Ta alloy has been recently demonstrated to be particularly promising and, in this paper, we investigate the origins of the observed improvements of the superconducting properties. Electron microscopy, Extended X-ray Absorption Fine Structure Spectroscopy (EXAFS) and Atom Probe Tomography (APT) characterization clearly show that, in presence of oxygen, both fine Nb_3_Sn grains and HfO_2_ nanoparticles form. Although EXAFS is unable to detect significant amounts of Hf in the A15 structure, APT does indeed reveal some residual intragrain metallic Hf. To investigate the layer properties in more detail, we created a microbridge from a thin lamella extracted by Focused Ion Beam (FIB) and measured the transport properties of Ta-Hf-doped Nb_3_Sn. *H*_*c2*_(0) is enhanced to 30.8 T by the introduction of Hf, ~ 1 T higher than those of only Ta-doped Nb_3_Sn, and, even more importantly the position of the pinning force maximum exceeds 6 T, against the typical ~ 4.5–4.7 T of the only Ta-doped material. These results show that the improvements generated by Hf addition can significantly enhance the high-field performance, bringing Nb_3_Sn closer to the requirements necessary for FCC realization.

## Introduction

Nb_3_Sn is being used to advance beyond Nb-Ti for the High Luminosity upgrade of the Large Hadron Collider ongoing at CERN, and it will be essential for the construction of the Future Circular Collider (FCC) in order to reduce or eliminate the cost of using High Temperature Superconductors (HTS)^1,2^. However, the FCC requirements in terms of non-Cu critical current density (non-Cu *J*_*c*_ > 1500 A/mm^2^ at 16 T and 4.2 K), residual resistivity ratio of the Cu stabilized (conductor RRR > 150 to maintain > 100 after cabling and winding) and filament diameter (d_eff_ < 20 µm) are very challenging and beyond the performance of presently available commercial Nb_3_Sn wires^3,4^. For this reason in the last few years there has been an increasing effort to improve the *J*_*c*_ performance of internal-tin (IT) and powder-in-tube (PIT) wires by investigating the phase evolution and by heat treatment optimizations^5–9^. Although a 36% *J*_*c*_ increase (*J*_*c*_(4.2 K,16 T) ~ 1300 A/mm^2^) was achieved in small filament diameter RRP (Restacked Rod Process) wires^8^, other approaches have also been pursued. The most promising is the introduction of artificial pinning centres by internal oxidation of the Nb alloy to form oxide nanoparticles, a technique which was first developed at GE (General Electrics) in 1994 using Nb-Zr to form ZrO_2_^10,11^, and which was recently re-examined using SnO_2_ as oxygen source^12,13^. Despite a clear shift to higher field of the pinning force maximum caused by the introduced pinning centres, adding only Zr causes a suppression of the irreversibility field *H*_*Irr*_ (and upper critical field *H*_*c2*_), clearly an undesirable feature for a high-field superconductor. In order to overcome this limitation, ternary alloys, such as Nb4Ta1Zr and Nb4Ta1Hf (atomic %) were developed to combine both the *H*_*c2*_ enhancing property of Ta with the pinning enhancing feature of Zr or Hf oxide nanoparticles^14^ (a similar study was conducted at the same time using NbTaZr alloy^15^). Although Hf had been previously used for Nb_3_Sn^16^, it had never been combined with Ta or with oxides. Our wires were made with a Nb-alloy rod surrounded by Cu-Sn powders (either with or without additional SnO_2_) introduced into Ta/Cu tubes. The best A15 performance was obtained with the Nb4Ta1Hf alloy with similar *J*_*c*_ and pinning properties either with or without SnO_2_. The pinning force (*F*_*p*_) maximum, *H*_*Max*_, evaluated by magnetic characterizations was observed at ~ 5.8 T in the TaHf-Nb_3_Sn sample, significantly higher than in Ta-Nb_3_Sn, where typically *H*_*Max*_ is at ~ 4.6 T. Moreover, the magnetization-estimated layer *J*_*c*_ (16 T) reached 3700 A/mm^2^ which, translated to a typical RRP layout, would lead to a non-Cu *J*_*c*_ of 2230 A/mm^2^, about 50% higher than the FCC specifications. Because of the wire design, transport measurements could not be directly performed on these samples and no *H*_*c2*_ characterization was carried out.

To better understand the origin of the enhanced pinning performance, in this study we directly investigated the superconducting properties of TaHf-Nb_3_Sn sample heat-treated at 550 °C/100 h + 670 °C/100 h by in-field transport measurements of the resistive transition to determine *H*_*c2*_(*T*), *H*_*Irr*_(*T*) and the field dependence of the pinning force *F*_*p*_(*H*). FESEM (field emission scanning electron microscopy) microstructure characterization was performed on fractured TaHf-Nb_3_Sn samples after different heat treatments and on a commercial Ta-doped RRP wire to compare the grain sizes. Extended X-ray Absorption Fine Structure Spectroscopy (EXAFS) characterization was carried out to evaluate where the Ta and Hf dopants are located in the Nb_3_Sn structure, whereas Atom Probe Tomography (APT) was used to provide local chemical composition, identifying also segregants and/or particles.

## Results

**Figure 1:**
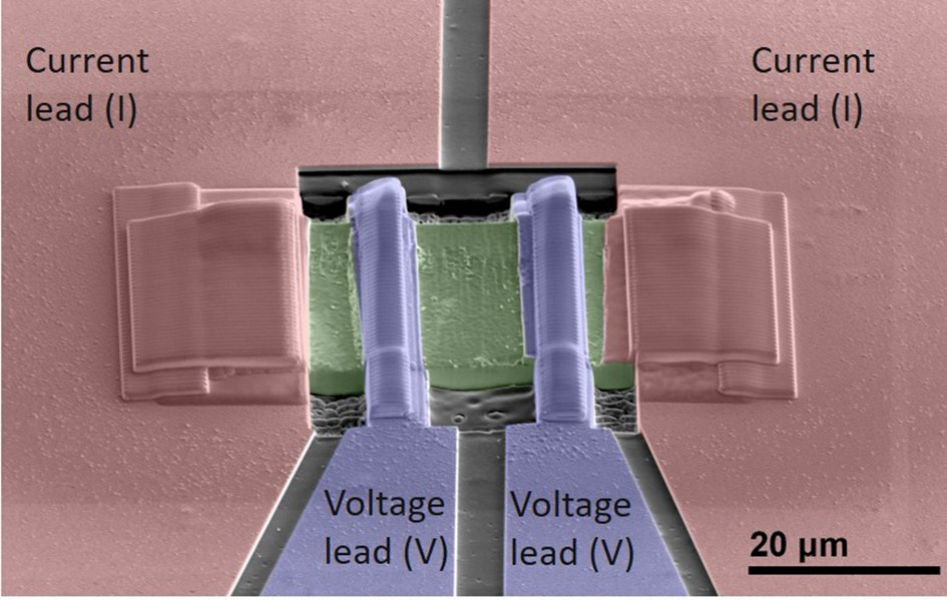
SEM image of Ta-Hf-doped Nb_3_Sn microbridge. False-coloured SEM image of a thin lamella (green) extracted by focused ion beam (FIB) from a Ta-Hf-doped Nb_3_Sn sample. The FIB’ed lamella is mounted on a MgO substrate. The current (pink) and voltage (blue) leads for transport measurements were deposited using a Gas Injection System (GIS).

**Figure 2:**
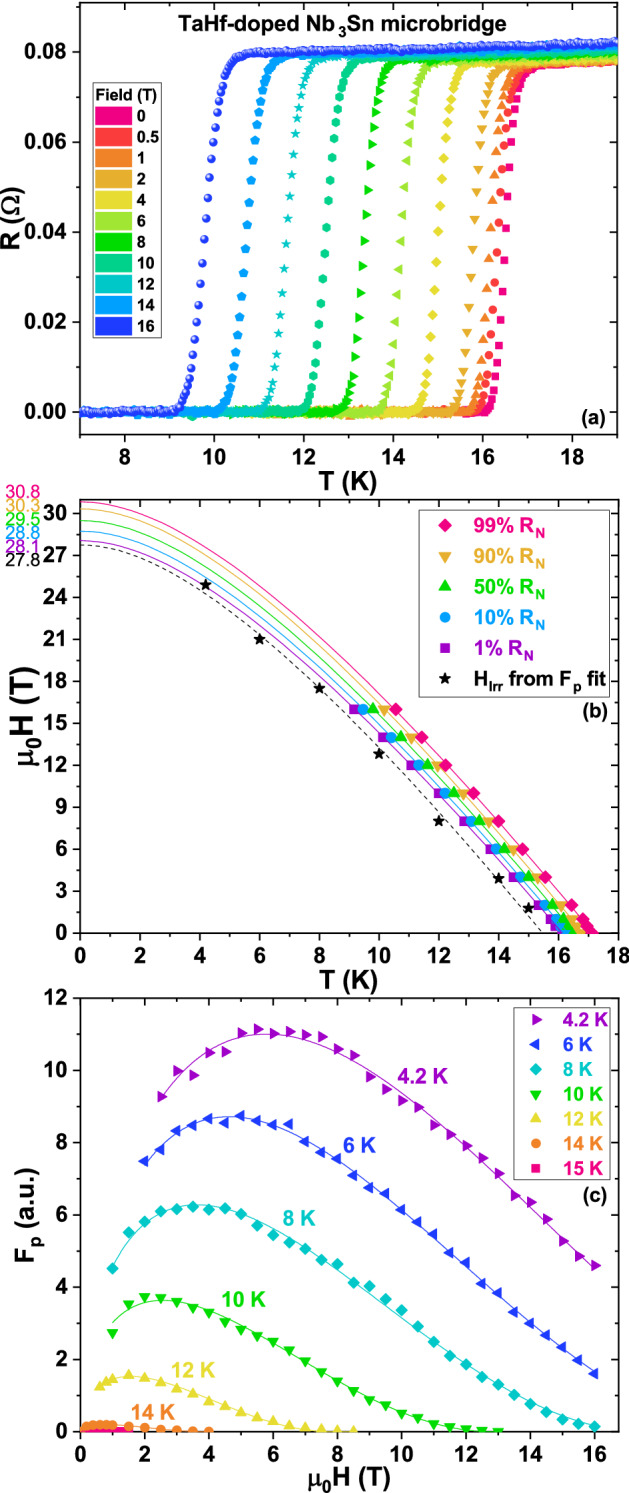
Transport characterizations of Ta-Hf-doped Nb_3_Sn microbridge. Superconducting characterization of a Ta-Hf doped Nb_3_Sn sample. (**a**) Resistive transitions at field up to 16 T. (**b**) *H*_*c2*_ (*H*_*Irr*_) versus temperature determined by different resistive criteria (see text) from (**a**) and from the fits in (**c**). (**c**) *F*_*p*_ versus field data at different temperatures with fitting curves (see text).

To overcome the limitation of the inductive characterization in our earlier evaluation of the superconducting properties, a microbridge for transport measurements was fabricated from the A15 layer of the wire heat treated at 670 °C. This microbridge allowed us to directly investigate the effect of the Hf addition on *H*_*c2*_ and to verify the shift towards high field of the *F*_*p*_ maximum. To fabricate the microbridge (shown in Fig. 1), a thin A15 lamella was sculpted out by Focused Ion Beam (FIB) and mounted on a MgO substrate. Metal current and voltage leads were then deposited by Gas Injection System (GIS) in the FIB. Some Ga ion implantation is expected during FIB bombardment (30 kV), producing a thin amorphous layer at the sample surface and with a maximum Ga penetration of the order of few tens of nm. This low level of Ga should not impact the superconducting properties of the sample (see “Methods”). The effective cross-sectional area of the Nb_3_Sn lamella could not be accurately measured: in fact, because of the brittle nature of the A15 phase, microfractures could easily be introduced when the FIB’ed lamella was extracted from the rest of the sample by the ex-situ micromanipulator. Although these types of damage did not prevent transport characterizations, they did prevent the evaluation of resistivity and *J*_*c*_ of the microbridge. Therefore, the transport characterizations were performed in 16 T QD-PPMS (equipped with external instruments) to obtain R(T,H) curves and to determine *H*_*c2*_ and *H*_*Irr*_. The resistive data, reported in Fig. 2a, show a very low noise level of few nV, corresponding to the intrinsic limit of our set-up, indicative of the high quality of microbridge and contacts. The transitions remained very sharp with almost no broadening when high field was applied, confirming the homogeneity of the sample. The curves were analysed with different resistive criteria, as shown in Fig. 2b, to estimate *H*_*c2*_ (typically estimated at 99 or 90 of the normal state resistance, R_N_; sometime at 50%) and *H*_*Irr*_ (typically estimated at 10 or 1% of R_N_). All datasets were fitted with the dirty-limit Werthamer, Helfand, and Hohenberg (WHH) model^17^ in order to estimate the zero-temperature values. Despite a slightly suppressed *T*_*c*_, *H*_*c2*_ in this Ta-Hf-doped wire is higher than that measured in only Ta-doped samples. In fact, in a wide high-field characterization campaign on Ta-doped Nb_3_Sn samples Godeke et al*.*^18^ estimated in a similarly heat treated sample (675 °C/64 h) a µ_0_*H*_*c2,99%*_(0) ~ 29.7 T , whereas in our TaHf-sample we found µ_0_*H*_*c2,99%*_(0) ~ 30.8 T, 1.1 T higher than in the Ta-case.

Despite the uncertainty in the lamella cross-section and the small surface area of the contacts in this microbridge, we were able to perform I-V characterizations and to investigate the profile of pinning force curves at different temperature between 4.2 and 15 K and up to 16 T (Fig. 2c). These data show that *F*_*p*_ maximum, *H*_*max*_, at 4.2 K is at about 6 T (the value estimated by magnetization was of 5.8 T)^14^, confirming the shift towards higher field with respect of typical Ta-doped value (~ 4.6–4.7 T). All *F*_*p*_(H) curves in Fig. 2c are well reproduced taking into account two pinning mechanisms, by grain boundaries (GB) and point defects (PD)^19^, using the equation ${F}_{p}\left(H\right)={A}_{GB}{f}_{GB}\left(\frac{H}{{H}_{Irr}}\right)+{A}_{PD}{f}_{PD}\left(\frac{H}{{H}_{Irr}}\right)={a}_{GB}{f}_{GB,norm}{f}_{GB}(\frac{H}{{H}_{Irr}})+{a}_{PD}{f}_{PD,norm}{f}_{PD}(\frac{H}{{H}_{Irr}})$, where ${f}_{GB}\left(h\right)={h}^{0.5}{(1-h)}^{2}$ and ${f}_{PD}\left(h\right)=h{(1-h)}^{2}$ (${f}_{GB,norm}$ and ${f}_{PD,norm}$ are the factors normalizing ${f}_{GB}$ and ${f}_{PD}$ to their maxima). Insulating oxide nanoparticles are a typical provider of point defect pinning sites. At 4.2 K the PD contribution is significant with ${A}_{PD}\sim 0.57{A}_{GB}$ (${a}_{PD}\sim 0.3{a}_{GB}$), but it is sharply suppressed with increasing temperature, becoming negligible at 10 K. Since the pinning efficiency is maximized when the defect sizes are about twice the coherence length, this loss of point pinning efficiency with increasing temperature suggests that a dimensional cross-over occurs at about 10 K, leaving only the grain boundaries as effective pinning centres at higher temperature. The *F*_*p*_(H) curve fits allow an estimation of the *H*_*Irr*_ values, shown in Fig. 2b. As for the *H*_*c2*_ data, they are fitted using the WHH model: the estimated *H*_*Irr*_(0) is 27.8 T, close to the value estimated from the R(T,H) measurements.

**Figure 3:**
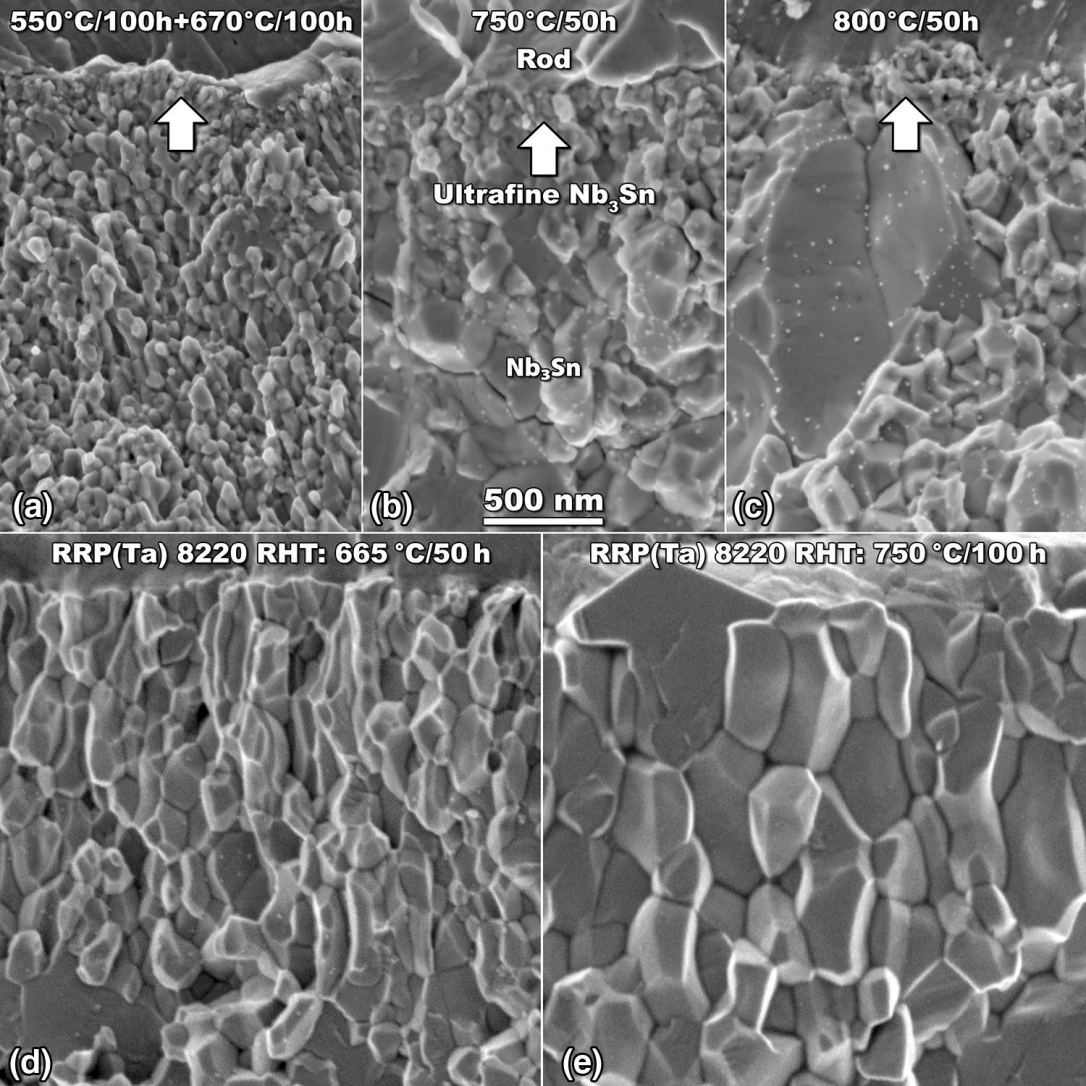
SEM characterization of Ta-Hf-doped Nb_3_Sn compared with standard Ta-doped Nb_3_Sn. Fractographs of Nb_3_Sn grains after heat treatments at different temperature for Ta-Hf-doped samples made with home-made Nb4Ta1Hf special alloy (**a**–**c**) and for Ta-doped RRP wire made with industrial Nb4Ta (**d**,**e**).

To understand the microstructural origin of the high field shift of *H*_*max*_, we used fractographs of the Nb_3_Sn layer for the Ta-Hf-doped wire reacted at 670 °C. Figure 3a reveals very small grains, with an average size of less than 70 nm, produced in a thick A15 layer (typical grain size in Ta-only doped Nb_3_Sn is 100–150 nm)^20^. However, no obvious particle or defect was observed that could explain the origin of the point defects. To investigate the evolution of the Nb_3_Sn microstructure in presence of both Ta and Hf, we also performed very high temperature heat treatments at 750 and 800 °C. Such high temperature heat treatments are, in general, undesirable because they are more difficult to use for wind-and-react magnets and because they cause grain growth, which is detrimental for the pinning performance. However, in this case the grain growth can be useful because it allows us to compare the difference in Nb_3_Sn grain growth depending on the dopants and to identify nanoparticles. Figure 3b,c show additional fractographs at 750 and 800 °C for the Ta-Hf-doped A15 layer compared with the typical microstructures in Fig. 3d,e of a RRP wire made with standard Nb4Ta alloy and reacted at 665 and 750 °C, respectively. Although the Ta-Hf-doped Nb_3_Sn grains grow with increasing HT temperature up to 800 °C (Fig. 3b,c), their sizes are still limited and very fine grains are still present near the unreacted alloy rod as a result of the delayed recrystallization of the Nb-4Ta-1Hf alloy compared with Nb-4Ta^14,21^. In fact, Fig. 3d,e shows the much larger A15 grains obtained in a Ta-doped A15 after the 665 and 750 °C reactions. Another important finding is that, although it is not possible to distinguish HfO_2_ nanoparticles in the 670 °C case, where the A15 grains are extremely small, they are obvious after the 750–800 °C HTs of the Ta-Hf wire, where the nanoparticles are clearly visible decorating the exposed grain boundaries. This result and the presence of a significant point pinning contribution in the *F*_*p*_ analysis suggests that the nanoparticles are also present after the 670 °C HT but that they are too small or too similar in size to the A15 facets to be distinguished.

**Figure 4:**
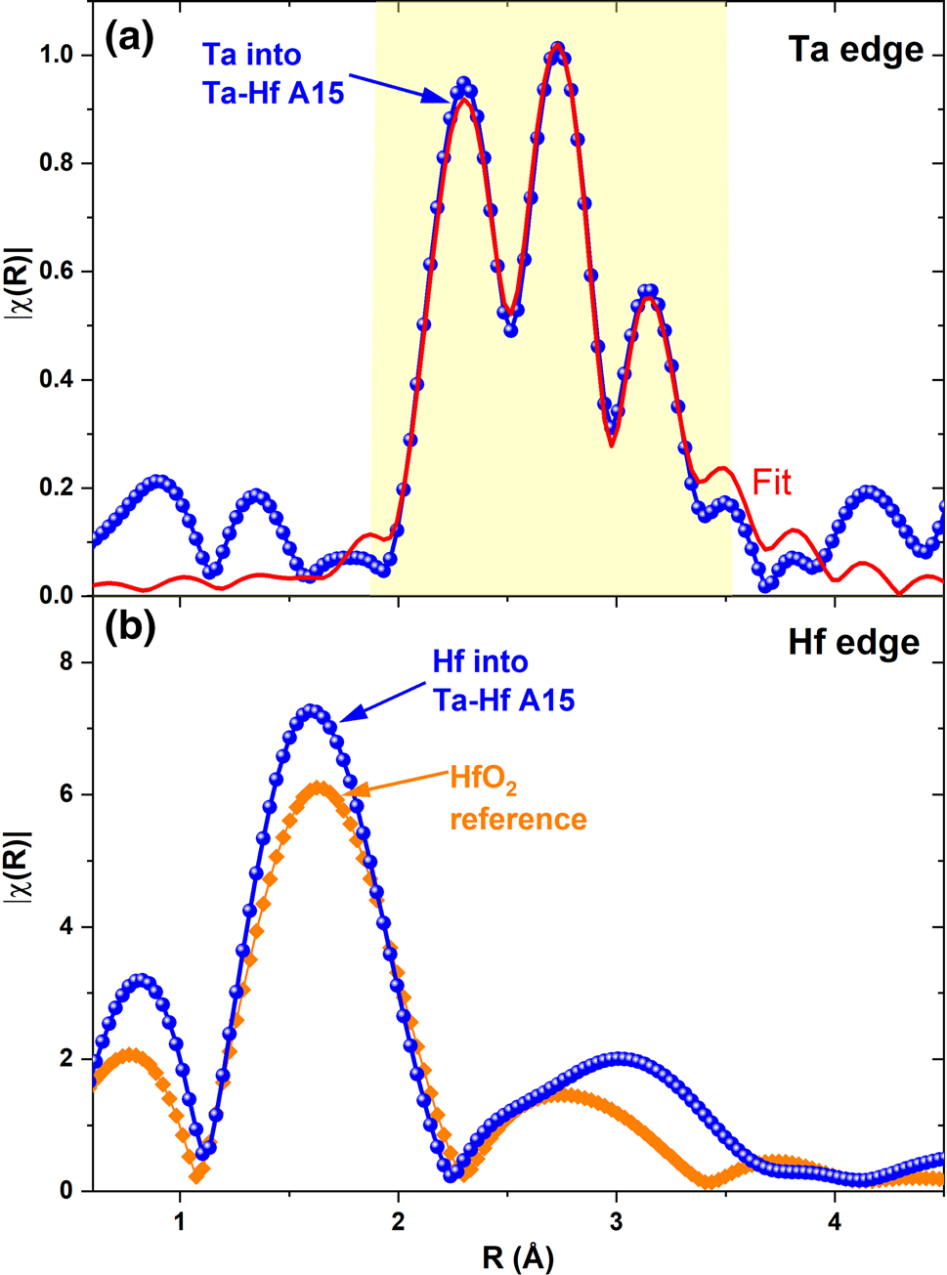
EXAFS characterization of Ta-Hf-doped sample. Fourier transforms of the *k*^*2*^ weighted χ(*k*) data for the (**a**) Ta L_3_ and (**b**) Hf L_3_ edges of the EXAFS characterizations of the Ta-Hf-doped Nb_3_Sn sample heat treated at 670 °C (blue dots) and of the reference HfO_2_ sample (orange diamond). In (**a**) the red line is the two-site fit for the Nb_3_Sn structure (fitting window of interest for Nb_3_Sn from 1.9 to 3.5 Å).

To clarify the nature of the point defects affecting the low-temperature pinning performance and to understand which role the two dopants play in the Nb_3_Sn layer, we also investigated our Nb4Ta1Hf-alloyed Nb_3_Sn sample using EXAFS. This technique allows us to determine whether a specific element is substituting for Nb and/or for Sn in the A15 structure or is located in another structure. In this case we focused our study on the EXAFS signals for both the Ta and the Hf edges produced by the A15 layer. The Fourier transform of the Ta-edge spectrum (Fig. 4a) reveals the typical 3-peak structure of the A15 phase due to majority Ta occupancy of the Nb sites (the three peaks are due to the three nearby coordination shells for the Nb site; occupancy of only the Sn site would produce one single peak in the central position)^22^. In fact, fitting these data shows that only a minor amount of Ta, ~ 12%, sits on the Sn site. Interestingly, this level of split site occupancy for Ta is compatible with that found for the Ta-doped RRP wire after similar heat-treatment^23^, suggesting that the presence of Hf does not affect the Ta occupancy behaviour. A similar analysis of the A15 layer for the Hf edge shows no clear indication of Hf being in the A15 structure (Fig. 4b): in fact, the major peak does not correspond to any of the peaks for the A15 phase, either the Nb or Sn site (compare with Fig. 4a). On the contrary, the Fourier transform of the spectrum obtained on the Ta-Hf-doped A15 layer corresponds to that obtained on the Hf edge of a reference HfO_2_ sample, suggesting that the Hf present in the Nb_3_Sn is mostly oxidized. It is important to note that, because of the proximity of the Hf edge to the Ta edge (they differ by ~ 320 eV), a quantitative analysis of the EXAFS signal of Hf cannot be performed, limiting our analysis to the qualitative description given above (see Supplementary Information and Figure S1 for details). Since the Fourier transform in Fig. 4b is dominated by the HfO_2_ signal, the presence of Hf in the A15 phase at levels obscured by the HfO_2_ component cannot be excluded by EXAFS.

**Figure 5:**
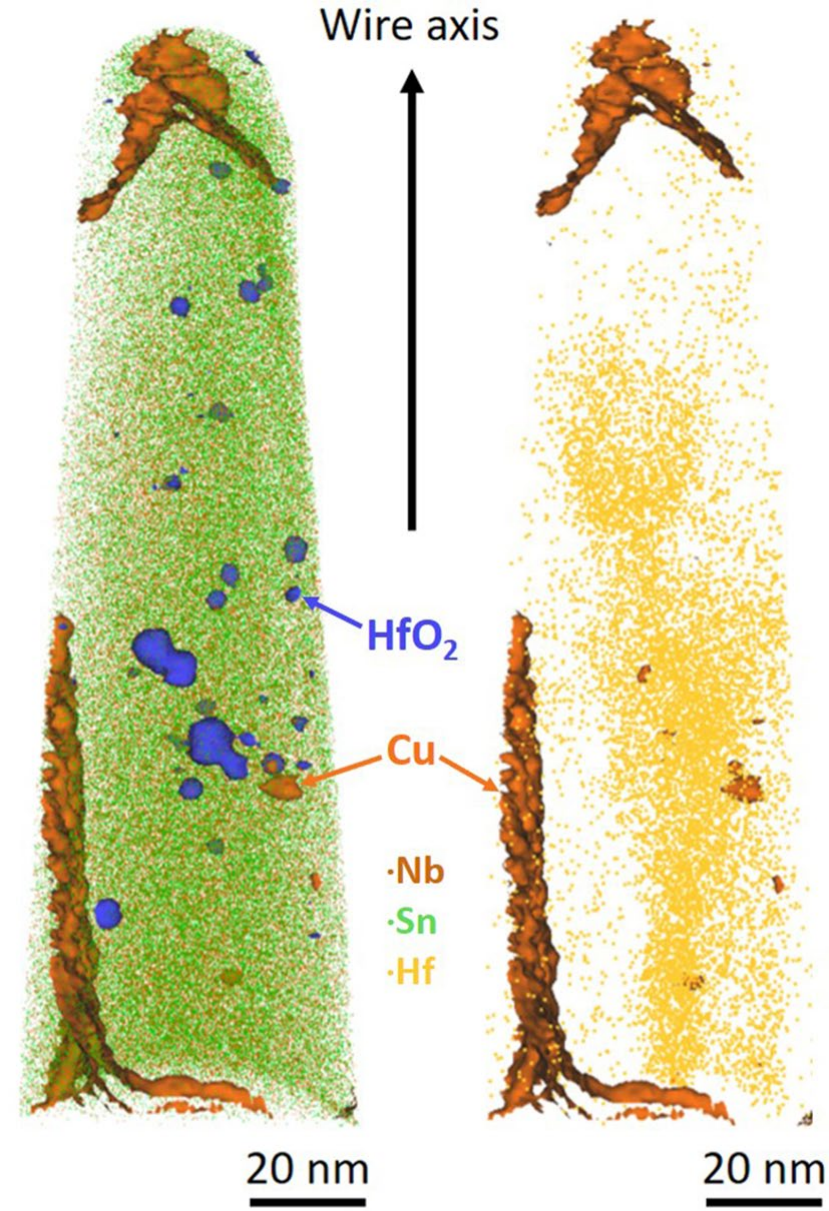
Atom Probe Tomography of Ta-Hf-doped Nb_3_Sn sample. Atom Probe Tomography of a needle-shaped sample (tip at the top of the figure) extracted by FIB from the A15 layer of the Ta-Hf doped Nb_3_Sn wire. The 4 at% copper iso-concentration surfaces in orange, identify the grain boundaries (and a few nanoparticles in the A15 grains). The blue iso-surfaces at 1.5 ionic% of HfO molecular species, in the left-hand image, identify the HfO nanoparticles; brown and green dots are Nb and Sn, respectively. In the right-hand image, the un-oxidized Hf species are represented by yellow spots.

To explore the possibility that very small HfO_2_ particles exist in the 670 °C sample, we employed Atom Probe Tomography analysis. This requires FIB’ing a needle-shaped sample out of the A15 layer^24^, and accelerating the atoms from the sample toward a detector^25^. Figure 5 shows a typical APT reconstruction of a volume of material containing several A15 grains. Copper is known to segregate to the GBs of Nb_3_Sn^26,27^ and so the 4 at% Cu iso-concentration surfaces reveal the location of the GBs (orange surfaces). We can identify separate peaks from metallic (unoxidized) Hf species and from HfO molecular ions in the mass spectra, and the distribution of these species is very different. The locations of HfO_2_ nanoparticles are clearly identified by the 1.5 ionic% iso-concentration surfaces for HfO species in Fig. 5 (blue surfaces in the left image), and their average diameter is ~ 3 nm with a density of ~ 5 × 10^22^ particles/m^3^. Clearly these HfO_2_ nanoparticles are not preferentially segregated at GBs but are found inside the A15 grain interiors. The right image of Fig. 5 shows the distribution of the un-oxidized Hf species, again within the A15 grains, but neither in the HfO_2_ particles nor in independent clusters. The combined EXAFS and APT results imply that, although the HfO_2_ nanoparticles are not clearly identifiable in the fractography of the sample heat treated at 670 °C (they are too small and fractography exposes only the GB interfaces), they are indeed present and likely the origin of the point-defect pinning contribution. The APT data also suggests that at least some of the Hf is not oxidized but is present as the solid solution in the A15 grains, although it gives no information on what site they occupy. The intragrain Hf concentration is estimated to be less than 0.1 at%, a small amount unlikely to be detectable by EXAFS.

## Discussion and conclusions

In this paper we investigated the origin of the enhanced performance of Nb_3_Sn made with a recently developed Nb4Ta1Hf alloy. The superconducting properties were measured in transport on a FIB-sculpted microbridge. They revealed an upper critical field *H*_*c2*_ that is about 1 T higher than the typical values obtained on standard Nb4Ta-alloyed Nb_3_Sn wires and can be attributable to the introduction of Hf. Furthermore, we observe a significant shift toward high field of the pinning force *F*_*p*_ maximum (~ 6 T versus 4.6–4.7 T in Ta-doped A15 samples). Deconvolution of the *F*_*p*_(H) curves clearly indicate a change in the pinning mechanisms, with a nanoparticle contribution being added to the typical grain-boundary pinning mechanism. Since this type of pinning mechanism is typically caused by the presence of point defects, such as nanoparticles, we investigated the microstructure by SEM imaging of fracture surfaces, and found that the A15 grains were significantly smaller than in the Ta-only case but we only saw a few nanoparticles. However, nanoparticles were more clearly observed in similarly prepared samples that underwent more extreme heat treatments. To better understand the role and effect of the Hf-addition on the A15 phase and its properties, we performed EXAFS characterizations that can reveal elemental substitution in the crystalline structure. We found that a significant amount of Hf is oxidized but, because of the closeness of the Ta and Hf absorption edges, no quantitative analysis of the presence of small amount of Hf in the A15 structure was possible. EXAFS, however, reveals that the addition of Hf does not affect the substitutional behaviour of Ta in the A15 structure, which is highly desirable because Ta is *H*_*c2*_-enhancing. Finally, we performed Atom Probe Tomography that unequivocally reveals the presence of a high concentration of HfO_2_ nanoparticles with an average diameter of about 3 nm, too small to identify with the other techniques, with a density of 5 × 10^22^ particles/m^3^. According to ref.^19^, the *A*_*PD*_*/A*_*GB*_ ratio obtained from the *F*_*p*_ fit can be expressed as *V*_*f*_/(1.16*⋅a⋅S*_*v*_), where *S*_*v*_ is the GB pinning surface area per unit volume projected in the direction of the Lorentz force, whereas *a* is the effective nanoparticle diameter and *V*_*f*_ is the fraction of flux-line lengths inside the nanoparticles. *S*_*v*_ ≈ 1/*d* (with *d* being the average grain size) and *V*_*f*_ should be between (*a*/*l*)^3^ and (*a*/*l*) in case of rigid and flexible flux lines, respectively (*l* being the average nanoparticle spacing), with *V*_*f*_ typically closer to (*a*/*l*)^3^. Using the microstructural information obtained by SEM (grain size) and APT (nanoparticle diameter and density), *A*_*PD*_*/A*_*GB*_ should range from ≈ 0.03 to ≈ 2.2, which is consistent with our experimental value of 0.57 with rather rigid flux lines.

APT study also shows presence of un-oxidized Hf within the A15 grains. It is presently unclear if this small amount of intragrain Hf, substituted into the A15 structure and presumably increasing the resistivity, is directly responsible for the *H*_*c2*_ enhancement or if other indirect factors are playing a role. Because Hf addition decreases the grain size and increases the grain boundary density, the Sn diffusion rate should be enhanced, possibly leading to a more Sn-rich, more homogeneous and better performing A15 phase. Another possibility is that the HfO_2_ nanoparticles strain the A15 structure, increasing the electron scattering and consequently *H*_*c2*_.

In conclusion, these findings on the intrinsic properties of Ta-Hf-doped Nb_3_Sn are promising from the point of view of the fabrication of conductors with improved high-field properties because of the combined effects of the high *H*_*c2*_ and the pinning-enhancing properties produced by the addition of Hf. The challenging task is now to reproduce such properties in a high-*J*_*c*_/high RRR conductor suitable for accelerator magnets.

## Methods

The Ta-Hf-doped Nb_3_Sn sample, made with Nb4Ta1Hf (atomic %) alloy, was realized as described in ref.^14^. The resulting doping levels into the A15 grains, far from the precipitates, were 3 at% of Ta and < 0.1 at% of Hf, as estimated by APT analysis. SEM characterization was performed in a Zeiss 1540 EsB Crossbeam Field Emission Scanning Electron Microscope (FESEM) using the in-lens secondary electron detector, whereas the microbridge fabrication were made in a Thermo Fisher Scientific Helios G4 UC FESEM equipped with Focused Ion Beam (FIB). To verify that Ga implantation does not significantly impact our sample properties, we estimated the depth of Ga penetration by simulation with the widely used Stopping and Range of Ions in Solids (SRIM) software^28^. We calculated a peak in the Ga concentration at about 10 nm from the surface and no Ga penetrating more than 35–40 nm. Since the thinnest sample dimension is 2.5 µm, about 99% of the sample cross-section should be Ga-free. Moreover, TEM of similar FIB’ed samples reveals that Ga is limited to an amorphous layer at the surface that, not having an A15 structure, has no effect of the superconducting properties. A Micro Support Axis Pro SS ex-situ micromanipulator was used for lifting out the FIB’ed lamella. Pt contacts were deposited using the ion/electron beam induced deposition and gas injection system (GIS) integrated into our FIB/FESEM. Transport characterizations were performed on the microcircuit in a Quantum Design 16 T physical property measurement system (PPMS) in which we used the four-contact measurement technique in maximum Lorentz force configuration. EXAFS characterizations and analysis were performed at the Advanced Photon Source beamline 20-ID at Argonne National Laboratory in conditions similar to those used in ref.^22^. The measurements were made in fluorescence using a bent Laue detector crystal with ~ 30 eV energy resolution to better separate the Hf and Ta fluorescence signals from the background Cu fluorescence. The x-ray beam size was 2 µm. See Supplementary Information for more details on EXAFS. Samples for the Atom Probe Tomography (APT) were created in a Zeiss 540 Crossbeam electron microscope using a Zeiss Nvision 40 FIB. The APT analysis was performed at a temperature of 60 K in a LEAP5000-XS in laser mode with a laser energy of 80 pJ.

## Supplementary Information

Supplementary Information.
