# Supplementary material for: Origin of the enhanced Nb3Sn performance by combined Hf and Ta doping
Source: Sci Rep. 2021 Sep 8;11:17845. doi: 10.1038/s41598-021-97353-w (PMC8426368; doi:10.1038/s41598-021-97353-w)
Supplement: Supplementary file 1 — Supplementary Information. [file 41598_2021_97353_MOESM1_ESM.pdf]

Supplementary Information

Origin of the enhanced Nb_3Sn performance by combined Hf and Ta doping

Chiara Tarantini[1], Fumitake Kametani[1,2], Shreyas Balachandran[1], Steve M Heald[3], Laura Wheatley[4], Chris R M Grovenor[4], Michael P Moody[4], Yi-Feng Su[1,5], Peter J Lee[1] and David C Larbalestier[1,2]

[1] National High Magnetic Field Laboratory, Florida State University, Tallahassee, FL 32310, United States of America

[2] Department of Mechanical Engineering, FAMU-FSU College of Engineering, Florida State University, Tallahassee, FL 32310, USA

[3] Advanced Photon Source, Argonne National Laboratory, Argonne, IL 60439, United States of America

[4] Department of Materials, Oxford University, Parks Road, Oxford, OX1 3PH, UK

[5] Now at Materials Science and Technology Division, Oak Ridge National Laboratory, Oak Ridge, TN 37831, USA.

* tarantini@asc.magnet.fsu.edu

EXAFS characterization. Figure S1 shows the normalized fluorescence as a function of the energy in the region including the Hf and Ta absorption edges, which differ by about 320 eV. To obtain the results shown in the main paper, the oscillations above the absorption edge (EXAFS range) are analyzed by Fourier transform of the spectrum. The typical analysis range is ΔE ~ 500 eV or more: in the Ta case the *k* range 2.5-13 $Å^{-1}$, corresponding to a ΔE ~ 620 eV, was used. Because of the proximity of the Ta edge to the Hf one, EXAFS of Hf was limited to the *k* range 2-8 $Å^{-1}$, corresponding to a ΔE ~ 230 eV: the limited range reduces the accuracy of the Fourier transformation, which still allow identification of the main structure in which Hf is included (HfO_2) but does not allow fitting to identify a possible underline presence of Hf in another structure (like the Nb_3Sn). Because of the very small amount of metallic Hf found by APT in the Nb_3Sn grains (<0.1 at% of the entire sample, i.e. <10% of the total Hf), the Fourier transform is dominated by the signal of the major structure in which Hf is present (HfO_2): Hf in the A15 structure would likely be near the detection limit by EXAFS even in the case of analysis of a typical energy range.

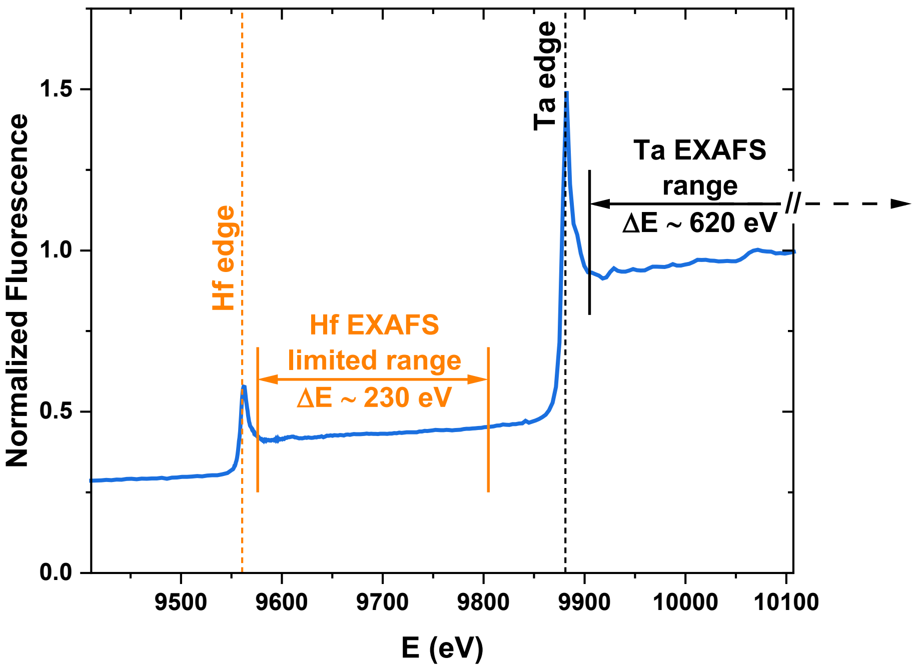

Figure S1. Normalized fluorescence as a function of the energy obtained for EXAFS characterization of the Ta-Hf-doped Nb_3Sn. Data obtained using a detector that does not discriminate the signals. Dashed lines indicate the positions of the Hf and Ta absorption edges. The energy ranges used for EXAFS analysis are also indicated in the figure.
